# Artificial Intelligence in the Management of Malnutrition in Cancer Patients: A Systematic Review

**DOI:** 10.1016/j.advnut.2025.100438

**Published:** 2025-05-05

**Authors:** Marco Sguanci, Sara Morales Palomares, Giovanni Cangelosi, Fabio Petrelli, Elena Sandri, Gaetano Ferrara, Stefano Mancin

**Affiliations:** 1 A.O. Polyclinic San Martino Hospital, Genova, Italy; 2 Department of Pharmacy, Health and Nutritional Sciences (DFSSN), University of Calabria, Rende, Italy; 3 School of Pharmacy, Polo Medicina Sperimentale e Sanità Pubblica “Stefania Scuri,” Camerino, Italy; 4 Faculty of Medicine and Health Sciences, Catholic University of Valencia San Vicente Mártir, c/Quevedo, Valencia, Spain; 5 Nephrology and Dialysis Unit, Ramazzini Hospital, Carpi, Italy; 6 IRCCS Humanitas Research Hospital, via Manzoni 56, 20089, Rozzano, Milan, Italy

**Keywords:** artificial intelligence, malnutrition, oncology, cachexia, machine learning, deep learning, nutritional assessment, body composition, clinical outcomes, cancer patients

## Abstract

Malnutrition is a critical complication among cancer patients, affecting ≤80% of individuals depending on cancer type, stage, and treatment. Artificial intelligence (AI) has emerged as a promising tool in healthcare, with potential applications in nutritional management to improve early detection, risk stratification, and personalized interventions. This systematic review evaluated the role of AI in identifying and managing malnutrition in cancer patients, focusing on its effectiveness in nutritional status assessment, prediction, clinical outcomes, and body composition monitoring. A systematic search was conducted across PubMed, Cochrane Library, Cumulative Index to Nursing and Allied Health Literature, and Excerpta Medica Database from June to July 2024, following the Preferred Reporting Items for Systematic Reviews and Meta-Analyses guidelines. Quantitative primary studies investigating AI-based interventions for malnutrition detection, body composition analysis, and nutritional optimization in oncology were included. Study quality was assessed using the Joanna Briggs Institute Critical Appraisal Tools, and evidence certainty was evaluated with the Oxford Centre for Evidence-Based Medicine framework. Eleven studies (*n* = 52,228 patients) met the inclusion criteria and were categorized into 3 overarching domains: nutritional status assessment and prediction, clinical and functional outcomes, and body composition and cachexia monitoring. AI-based models demonstrated high predictive accuracy in malnutrition detection (area under the curve >0.80). Machine learning algorithms, including decision trees, random forests, and support vector machines, outperformed conventional screening tools. Deep learning models applied to medical imaging achieved high segmentation accuracy (Dice similarity coefficient: 0.92–0.94), enabling early cachexia detection. AI-driven virtual dietitian systems improved dietary adherence (84%) and reduced unplanned hospitalizations. AI-enhanced workflows streamlined dietitian referrals, reducing referral times by 2.4 d. AI demonstrates significant potential in optimizing malnutrition screening, body composition monitoring, and personalized nutritional interventions for cancer patients. Its integration into oncology nutrition care could enhance patient outcomes and optimize healthcare resource allocation. Further research is necessary to standardize AI models and ensure clinical applicability. This systematic review followed a protocol registered prospectively on Open Science Framework (https://doi.org/10.17605/OSF.IO/A259M).

**Statements of Significance**

This systematic review highlights the transformative potential of artificial intelligence (AI) in oncology nutrition by demonstrating its superior accuracy in malnutrition detection, body composition monitoring, and personalized dietary interventions. Unlike previous studies, which focused on isolated AI applications, this work comprehensively evaluates AI-driven models across multiple clinical domains, emphasizing their integration into routine cancer care to enhance early detection, treatment personalization, and overall patient outcomes.

## Introduction

Cancer remains one of the leading causes of morbidity and mortality worldwide, with an estimated 20 million new cases and ∼9.7 million deaths reported in 2020, according to the Global Cancer Observatory [1]. The most prevalent cancer types include lung, breast, colorectal, and prostate cancer, accounting for a significant proportion of the global disease burden [2]. Advances in cancer treatment, including surgery, chemotherapy, radiotherapy, and targeted therapies, have improved survival rates. However, these interventions are often associated with substantial side effects that can profoundly impact patients’ nutritional status and overall quality of life [[3], [4], [5]].

Malnutrition is a prevalent and critical issue among cancer patients, affecting ∼40% to 80% of individuals, depending on cancer type, stage, and treatment methods [6]. Cancer-related malnutrition is often multifactorial, driven by tumor-induced metabolic alterations, treatment-related side effects, and decreased dietary intake due to symptoms such as cachexia, nausea, and dysphagia [[7], [8], [9], [10]]. A recent review has highlighted that this condition is a distinct manifestation of metabolic alterations, often coexisting with reduced food intake due to symptoms caused by the disease or its treatment (e.g., nausea, dysphagia, odynophagia, dysgeusia, etc.) [11].

The European Society for Clinical Nutrition and Metabolism has identified cancer-associated malnutrition as a major contributor to poor clinical outcomes, including increased morbidity, prolonged hospital stays, reduced treatment tolerance, and diminished survival rates [12].

Malnutrition frequently develops insidiously throughout the disease trajectory, becoming particularly evident in patients undergoing intensive treatment or in the advanced stages of malignancy [13]. The pathophysiology involves a complex interplay between systemic inflammation, metabolic dysregulation, and increased energy expenditure, leading to progressive muscle wasting and loss of functional capacity [14]. One of the most concerning manifestations of cancer-related malnutrition is sarcopenia, characterized by a progressive decline in skeletal muscle mass and strength [15]. Sarcopenia not only exacerbates physical impairment and treatment-related toxicity but also serves as an independent predictor of adverse clinical outcomes, including increased hospital readmissions and reduced overall survival [16].

In recent years, artificial intelligence (AI) has emerged as a transformative force in healthcare, offering innovative solutions to optimize patient management and clinical workflows [17]. AI-driven technologies, including machine learning (ML) and deep learning (DL), have demonstrated remarkable potential in enhancing diagnostic accuracy, personalizing treatment regimens, and improving resource allocation in hospital settings [18]. In the context of cancer care, AI applications have been successfully employed in medical imaging, genomics, and treatment response prediction [19,20].

Regarding malnutrition management, AI-based approaches present promising opportunities for early detection, risk stratification, and individualized nutritional interventions [21]. ML algorithms can analyze large datasets comprising clinical, biochemical, and imaging parameters, identifying high-risk patients with greater precision than traditional assessment methods [22]. Moreover, DL techniques, particularly convolutional neural networks and natural language processing, can facilitate the automated extraction of nutritional risk factors from electronic health records and medical imaging, providing clinicians with real-time, actionable insights [23]. By integrating AI into nutritional care, healthcare providers can adopt a more proactive and personalized approach, ensuring timely interventions to mitigate the deleterious effects of malnutrition and sarcopenia [24].

Given the high prevalence and clinical impact of malnutrition in oncology, there is an urgent need for more efficient and scalable strategies to identify and manage at-risk patients [25]. Traditional screening tools, such as the Malnutrition Universal Screening Tool, although widely used and valuable, could benefit from AI integration to enhance the efficiency and accuracy of nutritional assessments. AI-driven solutions can improve nutritional screening by automating data collection, detecting subtle patterns indicative of malnutrition, and facilitating early nutrition intervention through the use of predictive analytics [26].

Furthermore, integrating AI into existing prehabilitation protocols, such as Enhanced Recovery After Surgery (ERAS), holds significant promise [27]. By leveraging AI-powered analytics, clinicians can monitor patients’ nutritional status more accurately throughout the cancer care continuum, from diagnosis through preoperative adjuvant therapy to postsurgical recovery, thereby improving outcomes and reducing healthcare costs [28]. Addressing these gaps through AI-driven methodologies could lead to a paradigm shift in oncological nutritional care.

This systematic review aims to assess the role of AI in identifying and managing malnutrition in cancer patients, with a specific focus on its application in nutritional status assessment and prediction, as well as in clinical and functional outcomes and body composition and cachexia monitoring.

Although several reviews have examined AI in healthcare, oncology, or nutrition, this is the first systematic review specifically focused on the role of AI in the management of malnutrition in cancer patients. Unlike larger reviews, our work uniquely addresses how AI supports nutritional assessment, body composition monitoring, and cachexia management in oncology, providing a focused synthesis of current evidence in this clinically relevant domain.

The review synthesizes current evidence on AI-driven approaches, evaluates their diagnostic and prognostic accuracy, and explores their potential integration into routine oncological practice.

## Methods

### Review methodology and protocol registration

This systematic review was reported following the PRISMA guidelines [29]. The protocol for this review was registered in Open Science Framework, available at https://doi.org/10.17605/OSF.IO/A259M.

### Formulation of the research question

The research question for this review is: “*How does AI contribute to the identification and management of malnutrition in cancer patients?*” The formulation of this research question was guided by the PICO framework [30], which aids in structuring a clear and focused inquiry by addressing key components. In this context, the population (P) consists of cancer patients at risk of malnutrition; the intervention (I) includes AI-based approaches such as AI algorithms and predictive models; the comparison (C) refers to the standard of care, including clinical assessments and traditional nutritional screening tools; and the outcome (O) focuses on the improvement in the identification and management of malnutrition through AI-driven interventions.

### Search strategy

A systematic and comprehensive search was performed between June and July 2024 to identify relevant studies on the use of AI in identifying malnutrition in cancer patients. Key scientific databases, including PubMed, Cochrane Library, Cumulative Index to Nursing and Allied Health Literature (CINAHL), and Embase, were thoroughly searched. To ensure a broad and exhaustive analysis, additional gray literature sources and hospital-specific repositories were also explored, as these may provide valuable insights not included in traditional scientific publications. The search strategy used terms such as “cancer,” “malnutrition,” “artificial intelligence,” and “AI-based interventions,” along with their synonyms and related phrases. Boolean operators (AND, OR) were strategically applied to combine these terms to provide both a comprehensive and focused search (Supplemental Table 1 and 2). In the initial screening phase, 2 researchers independently reviewed all titles and abstracts retrieved from the database searches. Using EndNote 20 software (Clarivate), duplicates and irrelevant records were systematically excluded. Disagreements were resolved through discussion, and a third researcher was consulted if consensus could not be reached [31]. Full articles were obtained for studies deemed relevant based on pre-established eligibility criteria.

### Criteria and process

The inclusion criteria for this review comprised quantitative primary studies published in English that assessed AI-based interventions for the identification and management of malnutrition in cancer patients. Studies utilizing AI algorithms, ML models, or predictive analytics to detect malnutrition, monitor body composition changes, or optimize nutritional care in oncology settings were considered eligible. The AI-based interventions were evaluated in comparison to conventional approaches, including clinical judgment and standardized nutritional screening tools. Eligible studies focused on adult cancer patients at risk of malnutrition. Given the strong correlation between malnutrition and cachexia in this population, studies addressing cachexia-related outcomes were also included if they were aligned with the objectives of this systematic review.

The exclusion criteria encompassed secondary studies, such as narrative and systematic reviews, qualitative research, congress abstracts, book chapters, articles lacking accessible full texts, and publications deemed to be of low methodological quality. Studies published in languages other than English, those involving pediatric populations, noncancer patients, or interventions unrelated to AI were excluded. Furthermore, studies that solely examined nutritional screening tools without incorporating AI-based interventions were not considered for inclusion.

### Evaluation of risk of bias and methodological quality of studies

The risk of bias and the methodological quality of the included studies were assessed independently by 2 researchers. Any disagreements were resolved through consultation with a third researcher. To evaluate methodological quality, the Joanna Briggs Institute (JBI) Critical Appraisal Tools were used [32]. Studies were categorized based on their JBI scores: those with scores >70% were considered high quality, those between 50% and 70% as medium quality, and those <50% as low quality [33] (Supplemental Table 3).

### Assessment of evidence certainty

The certainty of the evidence was assessed using the framework established by the Oxford Centre for Evidence-Based Medicine (OCEBM) [34]. The OCEBM levels of evidence categorize research based on study design and methodology quality. Studies that include systematic reviews of randomized controlled trials (RCTs) and well-conducted individual RCTs are classified as Level 1 evidence. Studies based on expert opinion or lacking empirical support are classified as Level 5 evidence. Intermediate-level research, such as less rigorous RCTs, observational studies, or case series, are classified at levels 2, 3, and 4, respectively [35].

### Data extraction and synthesis

Data from the selected studies were extracted and summarized in tables, capturing key information such as the authors, year of publication, country, study design, population, type of AI intervention, outcomes, results, and assessment of quality/bias. The results are presented according to the objectives of the review through a narrative synthesis and summarized in figures and tables.

## Results

### Screening

**FIGURE 1:**
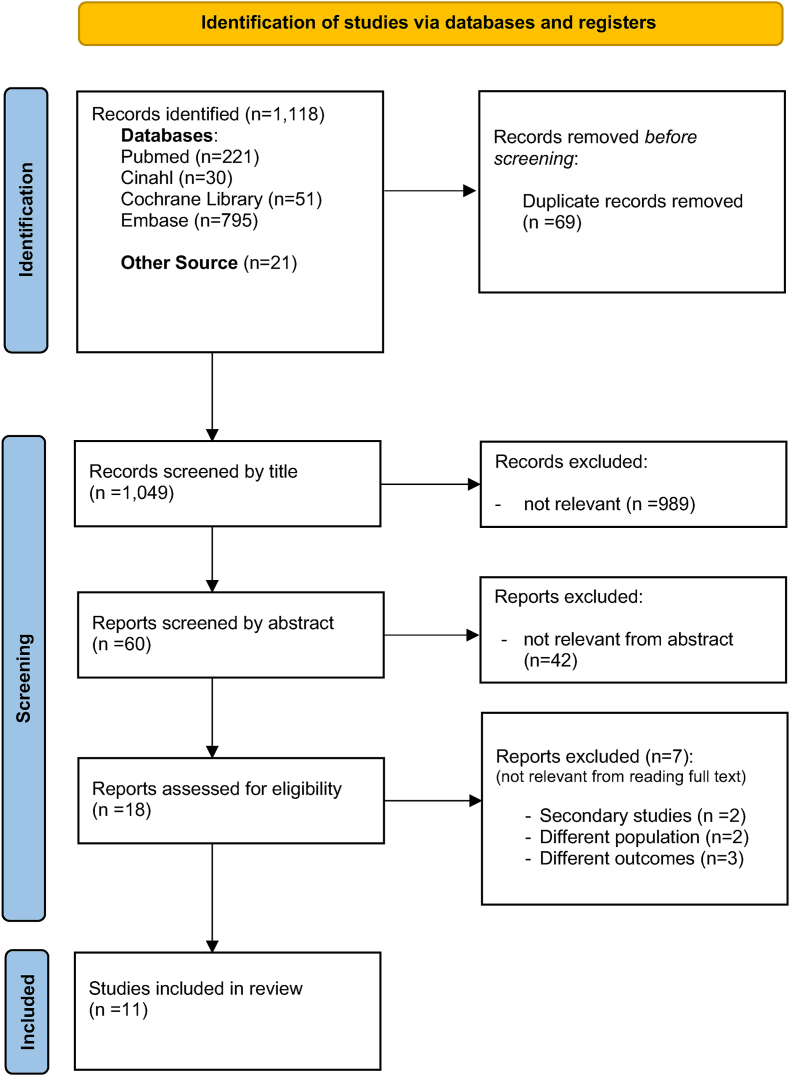
PRISMA flowchart.

A comprehensive search across multiple databases initially retrieved 1118 records: Cochrane Library (*n* = 51), PubMed-Medline (*n* = 221), CINAHL (*n* = 30), Embase (*n* = 795), and gray literature (*n* = 21). After removing 69 duplicates, 1049 records remained for further screening. A manual screening of titles eliminated 989 irrelevant articles, leaving 60 records for abstract screening. During this phase, 42 records were excluded due to being off topic and focused solely on conventional nutritional screening tools without integrating AI-based methods, resulting in 18 records for eligibility assessment. Of these, 7 were excluded for various reasons: secondary studies (*n* = 2), different population (*n* = 2), and different outcomes (*n* = 3). Finally, 11 studies were identified that met the inclusion criteria (Figure 1).

### Characteristics of the studies included

The included studies [[36], [37], [38], [39], [40], [41], [42], [43], [44], [45], [46]] originated from diverse geographical regions, with the majority conducted in China (54.5%) [36,37,43,45,46], followed by the United States (9.1%) [38], the United Kingdom (9.1%) [42], Australia (9.1%) [41], the Netherlands (9.1%) [39], and South Korea (9.1%) [44]. The study designs primarily included cohort (54.5%) and observational (45.5%) studies, covering a time range from 2021 to 2025 and comprising a total of 52,228 patients, with individual sample sizes ranging from 96 to 14,134 patients, addressing a broad spectrum of cancer subtypes.

The majority of studies demonstrated high methodological quality, as assessed through the JBI quality appraisal tools, with an average percentage of 83% (range: 72%–100%). The studies adhered to the standards of the Oxford Centre for Evidence-Based Medicine (OCEMB), ensuring a comprehensive evaluation and high validity of their findings. The evidence grade, ranging from 2 to 3, varied based on the study design (Table 1) [31,33,[36], [37], [38], [39], [40], [41], [42], [43], [44], [45], [46]].

**TABLE 1:** Characteristics of the included studies

Reference Country	Study design	Population	AI intervention	Standardized outcomes	Results	Quality/bias	Certainty level
Yin et al. [36], 2025 China	Multicentre cohort study	Cancer (*N* = 3767)	ML model to predict PRCC	Body composition and cachexia monitoring	AUC 0.887, sensitivity 0.859, specificity 0.812, accuracy 0.836	+++/Low	2
Wu et al. [37], 2024 China	Multicentre cohort study	Colorectal cancer (*N* = 4487) IG, *n* = 3365 CG, *n* = 1122	Random forest model	Nutritional status assessment and prediction	AUC 0.830, accuracy 0.775, sensitivity 0.835, specificity 0.742	+++/Low	2
Buchan et al. [38], 2024 United States	Observational study	Cancer (*N* = 3310)	AI-based nutrition assistant (Ina)	Clinical and functional outcomes	84% adherence, 82% QoL improvement, 88% symptom improvement	+++/Low	2
Daenen et al. [39], 2024 The Netherlands	Retrospective observational study	Lung cancer stage III (*N* = 140)	Deep learning (CycleGAN, CUT)	Body composition and cachexia monitoring	High segmentation accuracy (DSC 0.92–0.94), early detection potential	+++/Low	3
Costantino et al. [40], 2024 Italy	Retrospective observational cohort study	Oral/oropharyngeal cancer (*N* = 193)	ML prediction models	Nutritional status assessment and prediction	Accuracy 0.74–0.88, AUC 0.75–0.87	+++/Low	2
Kiss et al. [41], 2024 Australia	Cohort study	Cancer (*N* = 2494)	ML with GLIM criteria	Nutritional status assessment and prediction	Similar ML metrics with/without muscle mass (accuracy: 84% vs. 88%; sensitivity: 41% vs. 38%; specificity: 85% vs. 89%). Hospital admission: Almost identical ML metrics with/without muscle mass (accuracy: 77% vs. 77%; sensitivity: 29% vs. 29%; specificity: 84% vs. 84%).	+++/Low	3
Chung et al. [42], 2022 United Kingdom	Cohort study	Lung cancer (*N* = 76)	SVM	Clinical and functional outcomes	Misclassification errors <10%, effective early referral	+++/Low	2
Zhang et al. [43], 2022 China	Retrospective cohort study	Cancer (*N* = 702)	Decision tree, random forest	Nutritional status assessment and prediction	AUC 0.813, sensitivity 75.9%, specificity 73.3%	+++/Low	3
Chung et al. [44], 2023 South Korea	Cohort study	Gastric cancer (*N* = 4025) No CG	XGBoost	Body composition and cachexia monitoring	AUC 0.8237–0.8903, sensitivity 80%–86.96%, specificity 72%–74.60%	+++/Low	2
Yin et al. [45], 2021a, China	Observational cohort study	Cancer (*N* = 3998) IG, *n* = 2998 CG, *n* =1000	CART	Nutritional status assessment and prediction	AUC 0.964, accuracy 0.955, κ = 0.898	+++/Low	3
Yin et al. [46], 2021b, China	Observational cohort study	Cancer (*N* = 14,134)	K-means clustering	Nutritional status assessment and prediction	Moderate agreement with PG-SGA and GLIM criteria	+++/Low	3

Quality/bias according to Joanna Briggs Institute critical appraisal framework [31] and Morales Palomares et al. [33]: +++/Low; ++/Medium; +/High.

Abbreviations: AI, artificial intelligence; AUC, area under the curve; CART, classification and regression tree; CG, control group; CUT, contrastive unpaired translation; CycleGAN, cycle-consistent generative adversarial network; DSC, Dice similarity coefficient; GLIM, Global Leadership Initiative on Malnutrition; IG, intervention group; ML, machine learning; PG-SGA, Patient-Generated Subjective Global Assessment; PRCC, potentially reversible cancer cachexia; QoL, quality of life; SVM, support vector machine; XGBoost, extreme gradient boosting.

The evaluated outcomes were categorized into 3 overarching domains: nutritional status assessment and prediction, clinical and functional outcomes, and body composition and cachexia monitoring, providing a comprehensive overview of the integration of AI in the nutritional management of oncology patients.

### Nutritional status assessment and prediction

The assessment of malnutrition in oncology patients is crucial for optimizing treatment outcomes, given its significant impact on therapeutic tolerance, quality of life, and overall prognosis. The studies included in this category focused on the identification, classification, and prediction of malnutrition using AI, with a primary emphasis on diagnostic precision parameters such as sensitivity, specificity, and accuracy [37,40,41,43,45,46]. By integrating AI techniques, these studies aimed to enhance the objectivity and efficiency of nutritional screening and risk stratification, enabling timely and personalized interventions.

ML-based models have demonstrated considerable potential in predicting malnutrition risk by analyzing complex clinical and demographic data. Random forest models applied to colorectal cancer patients (*n* = 4487) achieved an area under the curve (AUC) of 0.830, with a sensitivity of 0.835 and specificity of 0.742, effectively identifying malnutrition even in the absence of traditional weight loss markers [37]. This approach highlights the ability of AI to process multidimensional datasets and capture subtle patterns that might be overlooked by conventional screening methods. Additionally, AI applications have proven valuable in predicting feeding tube dependence among oral and oropharyngeal cancer patients (*n* = 193), with accuracy values ranging from 0.74 to 0.88 and an AUC of 0.75 to 0.87. This supports clinical decision making by identifying patients requiring prolonged nutritional support [40]. These findings underscore the role of AI in facilitating targeted nutritional interventions and optimizing perioperative nutritional management strategies.

Two large-scale studies explored malnutrition classification and severity grading using different AI approaches. In the first study, a classification and regression tree (CART) model was applied to a cohort of 3998 cancer patients, achieving an AUC of 0.964, an accuracy of 0.955, and a kappa coefficient (κ) of 0.898, demonstrating substantial agreement with established malnutrition assessment tools, including the Patient-Generated Subjective Global Assessment (PG-SGA) and the Global Leadership Initiative on Malnutrition (GLIM) criteria [45]. In the second study, conducted on a larger cohort of 14,134 patients, a k-means clustering approach was used to stratify malnutrition severity, showing high concordance with PG-SGA and GLIM criteria [46]. Both studies highlight the scalability and adaptability of AI in refining malnutrition assessment, enabling the identification of malnourished patients with a high degree of precision in severity rating [45,46].

These studies therefore support a reflection on this AI model that could contribute to reducing the use of health resources once implemented, as it would allow the automation and acceleration of the malnutrition identification process.

The incorporation of phase angle measurements, a noninvasive marker derived from bioelectrical impedance analysis (BIA), into AI-based predictive models further enhanced malnutrition screening capabilities. A study conducted on hospitalized cancer patients (*n* = 702) demonstrated the efficacy of decision tree and random forest algorithms in predicting malnutrition risk, achieving an AUC of 0.813, with a sensitivity of 75.9% and a specificity of 73.3% [43]. These findings suggest that AI-driven strategies can integrate novel approaches to body composition assessment to improve risk stratification and guide personalized nutritional interventions.

**FIGURE 2:**
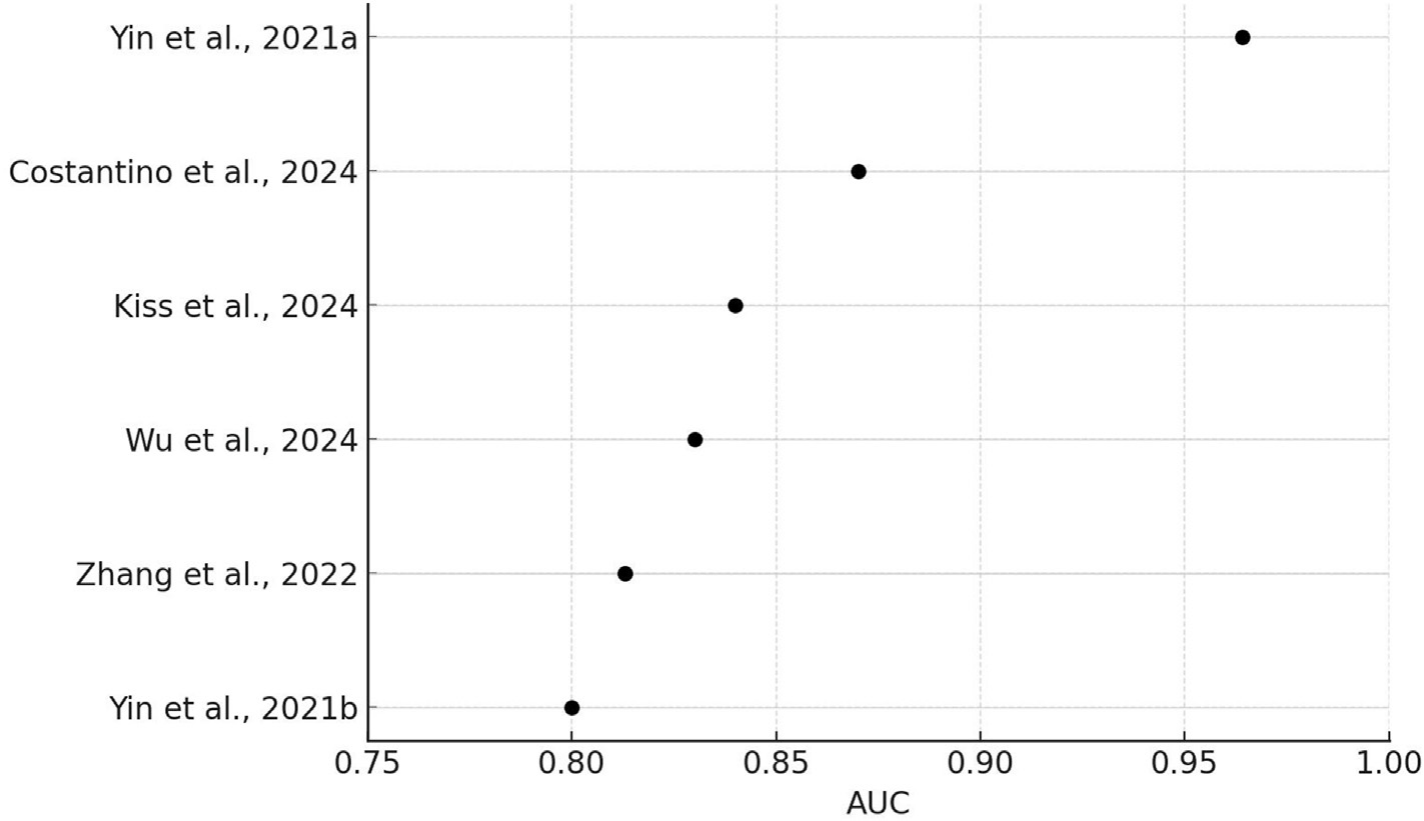
Performance of artificial intelligence (AI) models for nutritional status assessment. Forest plot showing area under the curve (AUC) values reported by the included studies evaluating AI models for nutritional status assessment in cancer patients. All models demonstrated good to excellent discriminative ability, with AUCs ranging from 0.813 to 0.964.

Collectively, the reviewed studies provide compelling evidence that AI-based models exhibit robust predictive capabilities in the assessment of malnutrition in oncology patients. The consistently high AUC values, coupled with favorable sensitivity and specificity metrics, indicate the potential of AI as an adjunct to traditional nutritional screening tools. By offering a more objective, data-driven approach, AI applications can streamline malnutrition assessment, enhance early detection, and facilitate tailored interventions to improve patient outcomes. A visual summary of AUC values and performance metrics of the AI models included in this domain is provided in Figure 2 to facilitate cross-study comparison.

### Clinical and functional outcomes

Malnutrition in cancer patients has profound implications beyond mere weight loss, affecting overall clinical outcomes, treatment response, and quality of life. The studies included under this outcome aimed to assess how AI-driven solutions can support clinical decision making by predicting functional deterioration, optimizing nutritional interventions, and enhancing patient-reported outcomes such as adherence to dietary plans and symptom management [36,38,42]. The AI technologies employed in these studies demonstrated the ability to provide actionable insights for both patients and clinicians, offering personalized recommendations based on real-time data and predictive analytics. Integrating AI into clinical workflows enabled the early identification of patients at risk of nutritional deterioration, allowing healthcare providers to intervene proactively. A notable contribution was the use of AI-based virtual dietitian systems, which demonstrated statistically significant improvements in dietary adherence (84%, *P* < 0.001), quality of life (82%, *P* = 0.002), and symptom management (88%, *P* < 0.001) among oncology patients undergoing multimodal treatment [38]. These systems integrated patient-specific data with ML algorithms to deliver personalized dietary recommendations, providing continuous support throughout the cancer care journey. Their implementation resulted in a notable improvement in patient-reported satisfaction: 93.6% of users expressed satisfaction with the platform (overall average rating: 4.0 out of 5). Furthermore, 98.4% of users found the tips helpful, and 97.3% appreciated the recipes, underscoring the platform’s meaningful clinical impact [38].

Another significant application of AI involved optimizing dietitian referrals in outpatient oncology settings. One study employed support vector machine models to classify cancer patients according to the PG-SGA, achieving an accuracy >90% and a misclassification error <10%. Key variables such as appetite and weight loss were identified, indicating that models with as few as 5 to 10 parameters could effectively predict nutritional risk [42]. These findings underscore the potential of AI to streamline clinical workflows, ensuring that high-risk patients receive timely interventions without overburdening healthcare resources. Furthermore, the system reduced the time to referral by an average of 2.4 d (*P* < 0.01) compared to standard clinical practice, thereby accelerating access to necessary nutritional support. The predictive capability of AI models extended to estimating the reversibility of cancer cachexia, a condition often resistant to standard nutritional interventions. The application of multilayer perceptron models achieved an AUC of 0.887 (95% CI: 0.862, 0.912; *P* < 0.001), with a sensitivity of 0.859 (*P* = 0.003) and specificity of 0.812 (*P* = 0.007), demonstrating their ability to distinguish patients likely to benefit from prehabilitative interventions [36]. By integrating diverse clinical and nutritional parameters, these models provided a deeper understanding of cachexia progression, supporting the development of personalized therapeutic strategies. AI-supported clinical workflows played a key role in improving functional status metrics, including better muscle mass retention, and demonstrated promising outcomes for patients with cachexia [36].

Overall, the reviewed studies highlight how AI can significantly enhance the management of malnutrition in cancer patients by improving clinical efficiency, enabling precision nutrition, and fostering patient engagement through digital interventions. The statistical robustness of AI-driven approaches, as evidenced by high AUC values, sensitivity, and specificity, suggests their potential to complement conventional clinical practices and improve long-term patient outcomes.

### Body composition and cachexia monitoring

The assessment of body composition and the monitoring of cancer cachexia progression are crucial components of oncological nutritional care. Traditional methods for evaluating muscle mass loss and fat depletion, such as computed tomography (CT) scans and BIA, are time-consuming and may lack precision in tracking disease-related changes. AI-based approaches have been developed to address these limitations by automating image analysis, predicting cachexia progression, and providing real-time feedback on body composition changes [36,39,44].

DL algorithms applied to imaging data have demonstrated high segmentation accuracy, with Dice similarity coefficients ranging from 0.92 to 0.94, enabling precise quantification of muscle and adipose tissue compartments [39]. These models facilitate the early detection of sarcopenia and cachexia, conditions strongly associated with poor treatment outcomes and increased mortality in oncology patients. AI-driven models for cachexia prediction have also yielded promising results in forecasting 5-year survival among gastric cancer patients, with AUC values ranging from 0.8237 to 0.8903, sensitivity levels between 80% and 86.96%, and specificity between 72% and 74.60% [44]. These findings suggest that AI tools can be effectively integrated into routine clinical practice to provide prognostic insights and guide individualized nutritional interventions aimed at mitigating cachexia-related complications. Furthermore, predictive modeling of potentially reversible cancer cachexia using ML achieved an AUC of 0.887, with sensitivity and specificity values of 0.859 and 0.812, respectively [36]. This highlights the utility of AI in distinguishing between reversible and refractory forms of cachexia, allowing clinicians to tailor interventions more effectively and allocate resources efficiently.

**FIGURE 3:**
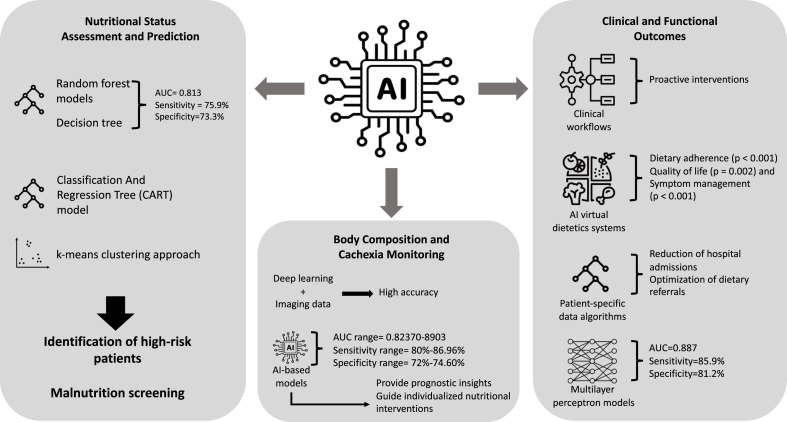
AI in the management of malnutrition in cancer patients. AI, artificial intelligence; AUC, area under the curve; CART, classification and regression tree.

Overall, the reviewed studies [36,39,44] demonstrate that the application of AI in body composition monitoring has the potential to transform oncological nutritional support by providing clinicians with accurate, real-time patient data. These advancements facilitate early intervention strategies, support the development of personalized nutrition plans, and ultimately contribute to improved clinical outcomes and enhanced quality of life for cancer patients. A graphical representation of the results is shown in Figure 3.

## Discussion

The aim of this systematic review was to evaluate the role of AI in identifying and managing malnutrition in cancer patients. The results obtained from the analysis of 11 studies that met the inclusion criteria highlight the enormous potential of AI, both currently and in the near future, for addressing malnutrition in oncology patients.

Innovative AI applications were identified in nutritional status assessment [37,40,41,43,45,46], body composition monitoring [36,39,44], and improving clinical and functional outcomes [36,38,42], reinforcing their role as transformative tools in nutritional oncology management. These findings align with previous research that emphasizes the ability of ML models to predict eating behaviors [47,48], estimate risk of developing eating disorders [49,50], and accurately detect malnutrition [[51], [52], [53]]. CART and random forest-based models achieved AUC values >0.80 in multiple studies [37,40,43,45], demonstrating high sensitivity and specificity.

The available literature confirms that AI-driven methods can quickly identify complex patterns in large volumes of clinical data, leading to efficient identification of patients at risk of nutrition complications during cancer treatment. AI tools have been shown to outperform traditional methods of clinical evaluation in other fields [[54], [55], [56]]; however, further research is needed to determine if the AI models outperform traditional malnutrition assessment methods in adults with cancer. Additionally, their concordance with established tools such as the PG-SGA [46] and the GLIM criteria [41, 46] strengthens their clinical utility and ability to integrate into existing healthcare workflows.

Although their integration is not without risks, ensuring transparency in models (Explainable AI), protecting sensitive patient data, and, most importantly, maintaining human oversight in decision making are critical for their effective use [57,58]. It is essential to recognize the indispensable role of healthcare professionals in medicine; AI should be a supportive tool, not a replacement. Physicians must retain control and make final decisions based on clinical judgment [59].

Accurate monitoring of body composition in cancer patients is crucial due to the insidious progression of cachexia and associated muscle loss [60]. In this context, DL algorithms applied to medical image segmentation have shown promising results, indicating high accuracy in quantifying muscle mass and adipose tissue [39]. This advancement overcomes the limitations of traditional methods, such as manual CT or BIA, which are more labor-intensive and subject to operator variability [61,62].

Furthermore, AI-based predictive models have proven useful in anticipating cachexia progression and distinguishing between reversible and refractory forms of this condition [36], the management of which through nutritional interventions remains an ongoing area of investigation [63]. These results enable the precise identification of patients who may benefit from targeted nutritional interventions, increasing the accuracy of at-risk patient detection and optimizing the allocation of available resources. By facilitating earlier and more personalized interventions, AI enhances the efficiency of clinical workflows and contributes to improved patient outcomes [64,65].

The integration of AI-based solutions into the nutritional care of oncology patients has also shown promising results in improving various clinical and functional parameters. Compared to standard nutritional care, AI-based virtual dietitian systems have shown potential and effective assessment and care capabilities. In one study, patients found the AI tool helpful, showing increased dietary adherence to 84% [38] accompanied by a significant increase in patient-reported satisfaction, reaching 93.6%. The use of AI has shown potential in another study in reducing treatment times by an average of 2.4 d, showing a predictive accuracy in identifying patients requiring nutritional support of >89%, with a misclassification rate of <0% [42].

The available data suggests that AI tools may have the potential to perform similarly to conventional methods of assessment and care, highlighting the potential of AI to enhance clinical management by providing personalized, real-time nutritional support [66,67], but comparison studies are needed. The optimization of healthcare resources driven by the increasing use of AI in medicine has far-reaching benefits. On one hand, it improves the quality of care by enabling faster and more accurate diagnoses and treatments, while early detection and prioritization of critical cases help prevent severe complications [68,69]. On the other hand, AI-driven healthcare strategies promote equitable resource distribution, allowing more patients to receive timely and appropriate care. Additionally, by supporting medical decision making through data-driven analyses, AI reduces the likelihood of misdiagnoses, enabling healthcare professionals to focus on complex cases that require human expertise [17,70].

### Strengths and limitations

One of the main strengths of this review lies in its broad and comprehensive approach, which has enabled the identification of AI applications across multiple aspects of oncological malnutrition management. The included studies span from initial nutritional assessment to advanced body composition monitoring, covering research conducted in different countries and utilizing varied methodologies.

The methodological quality of the included studies was high, with an average score of 83% according to the JBI quality appraisal tools, ensuring the validity of the results. The substantial sample size of 52,228 patients provides a robust foundation for drawing generalizable conclusions. Furthermore, the diversity in study designs, with a combination of cohort studies (54.5%) and observational studies (45.5%), allows for a comprehensive evaluation of associations and trends. Additionally, because the studies cover the period from 2021 to 2025, they provide updated and clinically relevant evidence.

The reliability and methodological rigor of this review are further strengthened by the use of recognized tools, such as the PRISMA criteria for systematization and the JBI standards for quality assessment. Another significant strength is the multidisciplinary composition of the research team, which includes experts from diverse fields, such as nursing, nutrition, and other healthcare disciplines. This diversity integrates both clinical and academic perspectives, ensuring that practical applications and theoretical implications are thoroughly addressed.

However, this review highlights several important limitations that should be addressed in future research. A major challenge is the substantial heterogeneity among the included studies, particularly in terms of AI algorithms, clinical settings, cancer types, outcome measures, and population characteristics. This variability hindered the feasibility of direct comparisons and precluded a meta-analysis. Moreover, the lack of standardization and transparency in the data used to train AI models, often derived from heterogeneous databases, raises concerns about the reproducibility and external validity of findings. While some models showed good concordance with established tools like the PG-SGA, inconsistent data sources may limit their reliability across different settings. Another notable limitation is the limited geographical diversity of the studies, the majority of which were conducted in high-income countries with advanced technological infrastructure. This restricts the generalizability of the results to low- and middle-income settings, where resource constraints may limit the implementation of AI-based healthcare tools.Finally, studies that focused solely on traditional nutritional screening methods without AI integration were excluded. While this aligns with the review’s objectives, it may have narrowed the perspective on nutritional care in oncology by omitting potentially relevant insights from non-AI-based approaches.

### Implication for clinical practice and future perspectives

The integration of AI-based technologies into clinical practice has the potential to improve quality of care, offering significant benefits in treatment personalization, healthcare resource optimization, and improved clinical outcomes. However, due to heterogeneity in access to oncological treatments and the diversity of available therapies, it is crucial to explore the most effective ways to implement these innovations into routine clinical practice.

Currently, many cancer patients undergo chemotherapy and radiotherapy in both outpatient and inpatient settings before becoming candidates for elective surgery. In this context, ERAS protocols play a crucial role in nutritional support, assisting patients through prehabilitation programs that have demonstrated significant benefits such as decreasing postoperative morbidity, reducing length of hospital stay, and Enhanced Recovery After Surgery, as reported in several studies [12,71].

A key challenge lies in integrating the findings of this systematic review to assess the potential of AI applied to prehabilitation programs for oncology patients undergoing surgery. The adoption of AI-based tools could provide advanced decision support, improve the personalization of therapeutic pathways, and enable more precise monitoring of patients’ nutritional status and response to prehabilitation protocols.

### Conclusion

The findings of this review highlight the enormous potential of AI in transforming the management of malnutrition in oncology patients. AI-based tools have demonstrated high effectiveness in identifying and classifying malnutrition risk, facilitating personalized nutritional interventions, and in some cases, improving key clinical outcomes.

The clinical impact of AI is also evident in functional improvements, such as higher adherence to personalized diets (84%) and a 23% reduction in unplanned hospitalizations. These results support the integration of AI-driven solutions into existing protocols, including prehabilitation and ERAS, allowing for more efficient and patient-centered nutritional management.

However, to maximize the impact of AI in clinical practice, it is essential to overcome key challenges identified in this review. These include the validation of AI technologies in diverse populations, the development of accessible algorithms for resource-limited settings, and the implementation of data standardization policies to enhance transparency and trust.

In summary, AI has the potential to redefine nutritional management in oncology by offering innovative, data-driven solutions. With proper implementation, these technologies could not only improve patients’ quality of life and survival but also optimize healthcare resources and promote more equitable and effective care across the oncology care continuum. The clinical acceptance of AI-driven tools will require significant investment in healthcare personnel training and the development of more interpretable and transparent AI models to ensure their seamless integration into routine clinical practice.

Future studies should focus on comparing AI-driven approaches with standard care practices to strengthen the evidence base before widespread implementation in diverse clinical settings. Additionally, research should evaluate the impact of AI on healthcare resource utilization (e.g., dietitian-patient ratios) to better understand how these technologies can be integrated into traditional care pathways.

The adoption of AI also entails economic considerations that may significantly burden healthcare systems. Therefore, future research should investigate the cost-effectiveness and financial sustainability of AI-based interventions, as these factors play a critical role in the feasibility and scalability of this emerging approach to care.

## Author contributions

The authors’ responsibilities were as follows – MS, SMP: performed conceptualization, methodology, and wrote the original draft, collaborating in review and editing process; FP, GF, ES: performed methodology, investigation, visualization and proofreading; SM: defined the scope and structure and supervised the review process; MS, SMP: provided an equal contribution as first author in design, writing, and final content of the manuscript; GF, SM: contributed equally as last author; and all authors: contributed substantial intellectual input to the manuscript in line with the International Committee of Medical Journal Editors (ICMJE) criteria for authorship, and read and approved the final manuscript.

## Data availability

The data that support the findings of this study are available on request from the corresponding author, upon reasonable request.

## Funding

The authors reported no funding received for this study.

## Conflicts of interest

The authors report no conflicts of interest.
