# Supplementary material for: Artificial Intelligence in the Management of Malnutrition in Cancer Patients: A Systematic Review
Source: Adv Nutr. 2025 May 5;16(7):100438. doi: 10.1016/j.advnut.2025.100438 (PMC12281439; doi:10.1016/j.advnut.2025.100438)
Supplement: Multimedia component 1 [file mmc1.docx]

**Artificial Intelligence in the Management of Malnutrition in Cancer Patients: A Systematic Review**

Author: Marco Sguanci RN, MSc, PhD

**Supplementary File**

**Supplementary Table S1:** Search Strategy - Databases

| Database | Search Terms | Mesh Terms | Keywords | Results |
| --- | --- | --- | --- | --- |
| PubMed | "Malnutrition"[MeSH] OR malnutrition OR "undernutrition" OR "nutritional deficiency" OR "nutritional status" OR "clinical malnutrition" AND ("Artificial Intelligence"[MeSH] OR "Machine Learning"[MeSH] OR "Predictive Models"[MeSH] OR AI OR "algorithm-based systems" OR "predictive analytics" OR "deep learning" OR "neural networks" OR "smart algorithms") AND "Neoplasms"[MeSH] | "Malnutrition"[MeSH], "Artificial Intelligence"[MeSH], "Machine Learning"[MeSH], "Predictive Models"[MeSH], "Neoplasms"[MeSH] | malnutrition, undernutrition, cancer, AI, predictive analytics, machine learning, deep learning, nutritional deficiency | 221 |
| Embase | 'malnutrition'/exp OR malnutrition OR 'undernutrition' OR 'nutrient deficiencies' OR 'nutritional assessment' OR 'clinical malnutrition' AND ('artificial intelligence'/exp OR 'machine learning'/exp OR 'data mining'/exp OR AI OR 'predictive modeling' OR 'neural networks' OR 'algorithm' OR 'deep learning') AND 'neoplasms'/exp | 'malnutrition'/exp, 'artificial intelligence'/exp, 'machine learning'/exp, 'data mining'/exp, 'neoplasms'/exp | malnutrition, undernutrition, nutrient deficiencies, cancer, AI, predictive modeling, deep learning, algorithm | 795 |
| CINAHL | (Malnutrition OR "Undernutrition" OR "Nutritional Deficiency" OR "Nutritional Status" OR "Clinical Malnutrition") AND ("Artificial Intelligence" OR "Machine Learning" OR "Predictive Modeling" OR "Deep Learning" OR "AI" OR "Algorithm-Based Systems") AND (Cancer OR "Neoplasms") | "Malnutrition", "Artificial Intelligence", "Machine Learning", "Predictive Modeling", "Cancer", "Neoplasms" | Malnutrition, undernutrition, cancer, AI, predictive modeling, deep learning, machine learning, algorithm-based systems | 30 |
| Cochrane Library | ("Malnutrition" OR "Undernutrition" OR "Nutritional Deficiency" OR "Nutritional Status" OR "Clinical Malnutrition") AND ("Artificial Intelligence" OR "Machine Learning" OR "Predictive Modeling" OR "Deep Learning" OR "AI" OR "Algorithmic Models") AND ("Cancer" OR "Neoplasms") | "Malnutrition", "Artificial Intelligence", "Machine Learning", "Predictive Modeling", "Cancer", "Neoplasms" | malnutrition, undernutrition, cancer, AI, predictive modeling, machine learning, neural networks, deep learning | 51 |

**TOTAL**: 1097

**Supplementary Table S2.** Google Scholar Search Strategy

| Search Terms | Keywords | Search Strategy | Results (selected) |
| --- | --- | --- | --- |
| "Artificial Intelligence" AND "Malnutrition" AND "Cancer" | AI, machine learning, cancer, malnutrition, nutritional deficiency, early detection, predictive analytics, oncology | "Artificial Intelligence" AND "Malnutrition" AND "Cancer" AND "Machine Learning" AND "Predictive Models" | 5 |
| "AI algorithms" AND "Malnutrition" AND "Cancer patients" | AI algorithms, cancer patients, nutritional assessment, early detection, machine learning, predictive tools | "AI algorithms" AND "Malnutrition" AND "Cancer patients" AND "Nutritional assessment" | 3 |
| "Artificial Intelligence in cancer" AND "Malnutrition identification" | AI, cancer, malnutrition identification, predictive modeling, machine learning, healthcare AI | "Artificial Intelligence in cancer" AND "Malnutrition identification" AND "Predictive Modeling" | 6 |
| "Artificial Intelligence" AND "Cancer" AND "Malnutrition detection" AND "Predictive analytics" | AI-based interventions, predictive analytics, malnutrition detection, cancer treatment, machine learning | "Artificial Intelligence" AND "Cancer" AND "Malnutrition detection" AND "Predictive analytics" | 4 |
| "Machine Learning" AND "Malnutrition" AND "Cancer diagnosis" | AI, machine learning, malnutrition detection, cancer diagnosis, early intervention, predictive tools | "Machine Learning" AND "Malnutrition" AND "Cancer diagnosis" AND "AI-based tools" | 6 |

**TOTAL**: 21

**Supplementary Table S3.** JBI Critical appraisal of cohort studies included

| **STUDY** | **ITEM 1** | **ITEM 2** | **ITEM 3** | **ITEM 4** | **ITEM 5** | **ITEM 6** | **ITEM 7** | **ITEM 8** | **ITEM 9** | **ITEM 10** | **ITEM 11** | **INCLUDE** | **Score**  **mean(%)** | **OCEBM**  **level** |
| --- | --- | --- | --- | --- | --- | --- | --- | --- | --- | --- | --- | --- | --- | --- |
| Yin et al., 2025, China^[35]^ | Y | Y | Y | U | N | Y | Y | Y | U | Y | Y | X | 81% | 2 |
| Wu et al., 2024, China^[36]^ | Y | Y | Y | U | N | Y | Y | Y | U | N | Y | X | 72% | 2 |
| Buchan et al., 2024, USA^[37]^ | Y | Y | Y | Y | Y | Y | Y | Y | Y | Y | Y | X | 100% | 2 |
| Daenen et al., 2024, Netherlands^[38]^ | Y | Y | Y | N | N | Y | Y | Y | U | Y | Y | X | 81% | 3 |
| Costantino et al., 2024, Italy^[39]^ | Y | Y | Y | Y | Y | Y | Y | Y | Y | N | Y | X | 91% | 2 |
| Kiss et al., 2024, Australia^[40]^ | Y | Y | Y | N | N | Y | Y | Y | U | Y | Y | X | 81% | 3 |
| Chung et al., 2024, UK^[41]^ | Y | Y | Y | Y | N | Y | Y | Y | U | Y | Y | X | 88% | 2 |
| Zhang et al., 2022, China^[42]^ | Y | Y | Y | U | N | Y | Y | Y | Y | Y | Y | X | 84% | 3 |
| Chung et al., 2023, South Korea^[43]^ | Y | Y | Y | N | N | Y | Y | Y | U | Y | Y | X | 81% | 2 |
| Yin et al., 2021a, China^[44]^ | Y | Y | Y | U | N | Y | Y | Y | Y | Y | Y | X | 84% | 3 |
| Yin et al., 2021b, China^[45]^ | Y | Y | Y | N | N | Y | Y | Y | U | N | Y | X | 75% | 3 |

*Legend: Y=Yes; N=No; U=Unclear; NA=Not Applicable; / Critical appraisal score according to Pimsen et al. (2022) / Items from JBI Critical appraisal checklist for Cohort Study: 1= Were the two groups similar and recruited from the same population? 2= Were the exposures measured similarly to assign people to both exposed and unexposed groups? 3= Was the exposure measured in a valid and reliable way? 4= Were confounding factors identified? 5= Were strategies to deal with confounding factors stated? 6= Were the groups/participants free of the outcome at the start of the study (or at the moment of exposure)? 7= Were the outcomes measured in a valid and reliable way? 8= Was the follow-up time reported and sufficient to be long enough for outcomes to occur? 9= Was follow-up complete, and if not, were the reasons to loss to follow up described and explored? 10= Were strategies to address incomplete follow-up utilized? 11= Was appropriate statistical analysis used?*
